# Recycling Old Antibiotics with Ionic Liquids

**DOI:** 10.3390/antibiotics9090578

**Published:** 2020-09-04

**Authors:** Cristina Prudêncio, Mónica Vieira, Seppe Van der Auweraer, Ricardo Ferraz

**Affiliations:** 1 Ciências Químicas e das Biomoléculas/CISA, Escola Superior de Saúde—Instituto Politécnico do Porto, Rua Dr. António Bernardino de Almeida 400, P-4200-072 Porto, Portugal; mav@ess.ipp.pt (M.V.); seppe.vanderauweraer@student.odisee.be (S.V.d.A.); 2 i3S-Instituto de Investigação e Inovação em Saúde, Universidade do Porto, 4200-135 Porto, Portugal; 3 Odisee University of applied sciences, Technology Campus Ghent, 26, 1000 Brussels, Belgium; 4 LAQV-REQUIMTE, Departamento de Química e Bioquímica, Faculdade de Ciências, Universidade do Porto, P-4169-007 Porto, Portugal

**Keywords:** ionic liquids, active pharmaceutical ingredients, antimicrobial agents, antibiotics

## Abstract

Antibiotics are considered one of the great “miracles” of the 20th century. Now in the 21st century in the post-antibiotic era, the miracle is turning into a nightmare, due to the growing problem of the resistance of microorganisms to classic antimicrobials and the non-investment by the pharmaceutical industry in new antimicrobial agents. Unfortunately, the current COVID-19 pandemic has demonstrated the global risks associated with uncontrolled infections and the various forms of impact that such a pandemic may have on the economy and on social habits besides the associated morbidity and mortality. Therefore, there is an urgent need to recycle classic antibiotics, as is the case in the use of ionic liquids (ILs) based on antibiotics. Thus, the aim of the present review is to summarize the data on ILs, mainly those with antimicrobial action and especially against resistant strains. The main conclusions of this article are that ILs are flexible due to their ability to modulate cations and anions as a salt, making it possible to combine the properties of both and multiplying the activity of separate cations and anions. Also, these compounds have low cost methods of production, which makes it highly attractive to explore them, especially as antimicrobial agents and against resistant strains. ILs may further be combined with other therapeutic strategies, such as phage or lysine therapy, enhancing the therapeutic arsenal needed to fight this worldwide problem of antibacterial resistance. Thus, the use of ILs as antibiotics by themselves or together with phage therapy and lysine therapy are promising alternatives against pathogenic microorganisms, and may have the possibility to be used in new ways in order to restrain uncontrolled infections.

## 1. Introduction

Initially in the fight against infectious diseases, heavy metals (arsenic, mercury, or bismuth) were used. Modern antimicrobial chemotherapy begins with the introduction in the 1930s of sulfonamides, and afterwards with the introduction, in the 1940s of penicillin (discovered in 1928 [1]) and streptomycin (1944) [2,3]. At this time, one died of syphilis, gonorrhea, pneumonia, and tuberculosis, among others, and the introduction of those antibiotics was considered “a miracle” [4]. However, resistance was quickly developed [3,5,6,7,8].

In recent years, the number of microorganisms with resistance has been growing at an almost exponential rate [9]. Also, the resurgence of pathologies that had formerly been almost eradicated justifies the relevance of this theme and the need to have new weapons or recycle the ones we already have [10]. The emergence of multiresistance presented by microorganisms or, in some cases, even extreme drug resistance (XDR), as observed in *Bacillus* Gram negatives, is one of the great challenges currently facing health professionals and the population in general [3,11]. Once bacteria demonstrate resistance to the first line antimicrobial agents, this necessitates the use of more costly antimicrobial agents of second and third lines [11,12]. Antimicrobial resistance threatens human lives and the effectiveness of health programs, and also results in greater costs [13]. Recently, antibacterial resistance has been described as a threat to world stability and the security of countries [11,14,15].

For the past 50 years, the “era of antibiotics” has been wonderful. However, we are now at risk in the “post-antibiotic era” of returning to the conditions encountered at the beginning of the 20th century, when the infections mentioned above were killing millions of people, and now, these and many others put the entire world population at risk [10,11,13,16]

Researchers are looking for new and improved drugs to treat bacterial infections [17]. Pharmaceutical researchers and manufacturers have focused on solid active ingredients in the form of tablets of powders, while liquids and transdermal forms are often neglected. Yet many solid drugs are too insoluble for our body to absorb and will not reach the bloodstream effectively. Meanwhile, ionic liquids (ILs) are an interesting class of compounds that could potentially overcome these delivery problems, although they are often being ignored [18]. The possibility to combine the ILs with other strategies makes them even more useful. One possible combined therapy to be explored could be the use of ILs in conjugation with phages or lysines. There are studies that describe the combined action of antibiotics and phages or lysines [19,20,21], but none used in combination with ILs. These new strategies could be a strong weapon especially against resistant strains of bacteria. In the present work, we focused on the potential of ILs by themselves as antimicrobial agents. 

## 2. Ionic Liquids and Active Pharmaceutical Ingredients (APIs)

### 2.1. Ionic Liquids

ILs are commonly defined as salts solely composed of cations and anions, with a melting temperature below 100 °C. They have a bulky and asymmetrical cation structure that lowers their melting point [22,23]. Although compounds fitting this definition were described at the beginning of the 20th century [24,25,26], only in recent decades have they become a topic of significant interest, with the number of articles involving ILs growing exponentially [24,27,28,29]. This topic has been the subject of several major reviews and books, which have dealt with a wide range of applications and aspects of ILs [30]. The range of applications has broadened remarkably in recent years, stimulating the industrial and academic interest in the applications of ILs in a broad array of life sciences, in particular the pharmaceutical applications of ILs [28,29,31,32].

ILs were first used as solvents and reagents in a wide range of pharmaceutical processes, and as reaction media in synthetic processes for new and known drugs [24,25]. Currently, ILs are used to form active pharmaceutical ingredients (APIs), taking advantage of their tunable chemical and biological activities, and are also used as part of distinct drug delivery systems for drugs with reduced bioavailability [31,33,34,35,36]. 

The tunable nature of ionic liquids reflects a fundamental difference between covalent and ionic bonds. A covalent bond is an interatomic bond in which a pair of valence electrons are shared between the interacting atoms. Ionic liquids, however, are composed of ions. These ions form ionic bonds through electrostatic interactions. This only occurs between atoms with a significant difference in electronegativity. Essentially, one atom with a low electronegativity donates a valence electron to the atom with a higher electronegativity [31,37]. Ionic interactions retain outstanding intrinsic potential for flexible and dynamic behavior, which can change the properties of ionic compounds. The dynamic and adjustable potential of ionic compounds can be powerful in the liquid phase [31]. 

The combination of the liquid phase and ionic character of bonding provides an opportunity to generate a diversity of ionic liquids with specific physical and chemical properties [38]. The nearly infinite combinations of suitable cations and anions allow for a wide range of modulation of the IL properties [39,40,41,42]. The anion is usually responsible for characteristics such as air and water stability, and the cation is usually responsible for the melting temperature and organic solubility [43].

ILs are known as “designer solvents”, since they allow physical tuning to obtain specific properties for a particular purpose or application [44]. Their extraordinary flexibility explains their wide area of applications [31,37]. Since there are a lot of possible anion and cation combinations, it is possible to tailor an IL molecule in order to achieve a desirable property [30]. Researchers can design a specific IL by choosing negatively charged small anions like [Tf_2_N]^−^, PF_6_^−^, or PF_4_^−^ and positively charged large cations like alkyl-imidazolium, alkyl-pyridinium, alkyl-pyrrolidinium, alkyl-phosphonium, or alkyl-morpholinium [31,37,45,46].

### 2.2. Pharmaceutical and Medicinal Applications of Ionic Liquids

**Figure 1:**
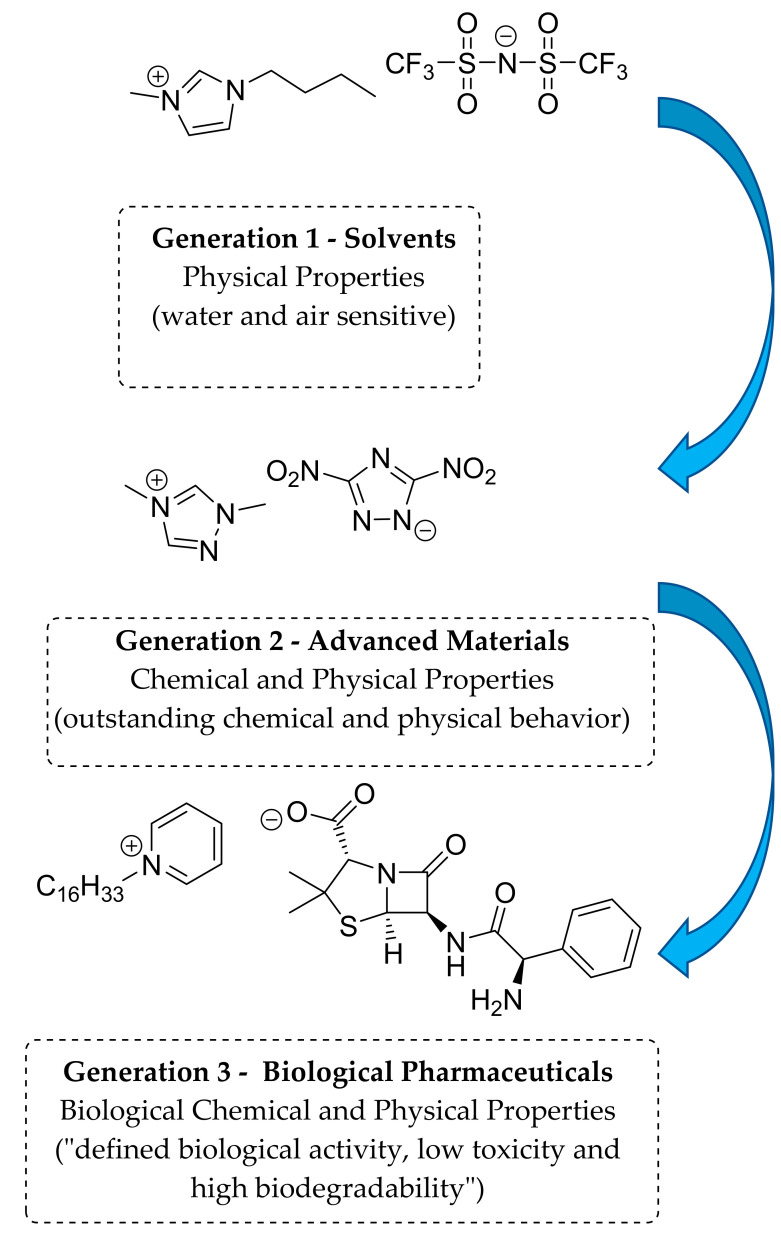
The three generations of ionic liquids (ILs) and their evolution adapted from Ferraz et al. [29].

The use of ILs in medicinals and pharmaceuticals is classified as the third generation of ILs [29,47]. There are three generations of ILs, depending of their structure and their properties [29,31,47] (Figure 1). The first generation is related to physical and chemical properties; the compounds were sensitive to water and air, and basically combined dialkylimidazolium and alkylpyridinium cations with metal halide anions [29,31,48]. The second generation is based on the potential to tune some physical and chemical properties, allowing the formation of “task-specific ionic liquids”, which can have applications as lubricants, energetic materials, and reaction solvents, among others. They are air- and water-stable; the most common cations include dialkylimidazolium, alkylpyridinium, ammonium, and phosphonium, whereas halides, tetrafluoroborate, and hexafluorophosphate are among the most common anions [29,31,49,50,51]. The third generation of ILs is related to active pharmaceutical ingredients (APIs), which are used to produce ILs with biological activity. API is the term used to refer to the biologically active component of a drug product. The third generation of ILs employs biodegradable and natural ions, such as choline and amino acids, or ions with known biological activities [26,29,31,47,49,52].

**Figure 2:**
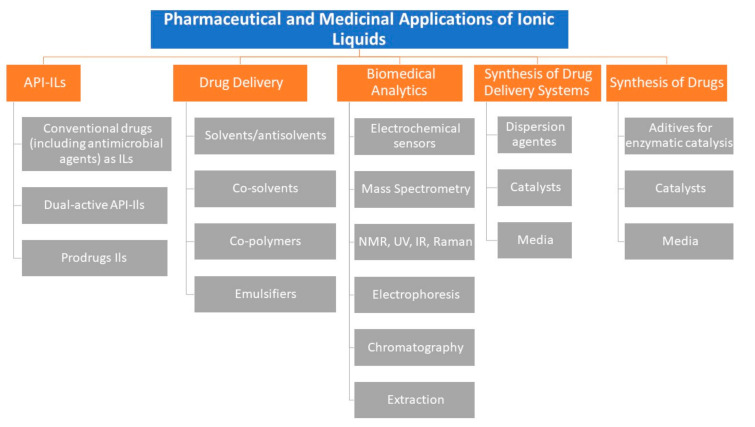
A summary of the many pharmaceutical and medicinal applications of ILs (adapted from Egorova et al. [31]). API: active pharmaceutical ingredient.

There are many pharmaceutical and medicinal applications of ILs (Figure 2) [28,31,32]. Nowadays, several fields of pharmaceutical industry already use ILs as components of drug or drug delivery systems and as solvents in drug synthesis [28,31,32]. ILs are being studied because of their biological activity [28], their biomedical applications [38], and their antimicrobial activity [40,46,53], and also in consideration of their environmental toxicity [37] and cytotoxicity against cancer cells [27,54], with some studies exploring the possibilities of IL biodegradation [55]. A summary of the many pharmaceutical and medicinal applications of ILs is provided in Figure 2.

### 2.3. Structure–Activity Relationships of Bioactive Ionic Liquids

Quaternary ammonium or pyridinium halides have been previously described with antimicrobial properties [40,46]. This characteristic together with the toxicity studies of new ILs led to the biological activity research of ILs and their application in the pharmaceutical and medical sciences and industries, which increased the attention paid to this type of IL [40,46]. Several types of cations, like imidazolium, pyridinium, pyrrolidinium, piperidinium, and ammonium, among others, have been shown to inhibit the growth of both environmental and clinically important pathogenic and nonpathogenic microorganisms [31].

As expected, there is some evidence that the alkyl chain is an important component, probably due to its tendency to disrupt the integrity of biological membranes [56]. This demonstrates that the length of the alkyl chain is very important to the antimicrobial activity. However, the manner in which the structure–activity relationship affects antimicrobial activity must be better understood [57,58,59,60]. Studies have shown that compounds with a short alkyl chain or a short functional side have weaker activity on several strains and types of microorganisms. One possible explanation could be the penetration of the long alkyl chain on the lipid membrane inducing structural damage [61]. 

One of the biggest concerns in medicinal chemistry is the bioavailability and biodisponibility of compounds [29], so these are important characteristic that should be studied with respect to ILs. Together with the interaction with water, these are important features that shape the biological activity of these compounds. As described by Kurnia et al., the arrangement between the cation and the anion can affect the bioavailability of the final compound [62]. In a study involving several cations containing butyl or isobutyl side chains and bis(trifluoromethylsulfonyl)amide anions, Kurnia et al. demonstrated that the water solubility of ILs was lower in the piperidinium ILs and increased in imidazolium ILs, with further increases in pyridinium, pyrrolidinium ILs, and imidazolium ILs. They suggested that this order was dependent on the water cavitation potential, which was influenced by the size and, to some degree, the aromaticity of the IL cation [31].

The length and the number of alkyl chains in the molecule is the main factor determining the antimicrobial activity of ILs [63]. The “ideal” length should be of 12 or 14 carbon atoms, as this range of lengths showed the highest antimicrobial activity, whereas aliphatic chains of over 16 or less than 10 carbon atoms showed a reduction in antimicrobial activity [46,63]. Another important feature of ILs is their chemical structure, in particular the presence of polar groups in the hydrophilic part of the IL. This will affect the antimicrobial activity significantly, as demonstrated for imidazolium, pyridinium, and pyrrolidinium type ILs [63,64].

### 2.4. ILs as Antimicrobial Agents

The production of ILs based on antibiotics could be tricky, due to the fact that antibiotics are fragile molecules [65,66]. They have high strain rings and they can be degraded with pH alterations [66]. The most traditional way to prepare ILs involves a metathesis reaction of an anion halide with an adequate alkaline salt. However, this reaction presents some inconveniences, such as its contamination with a small amount of halide ions [67], and this method can only be done with some bulky imidazolium and pyridinium ILs, as demonstrated by Cole et al. [68,69]. The ion exchange resin methods developed by Ohno et al. [70] (amberlite resin (in the OH form)) has been used in order to exchange halides (bromide or chloride) to the hydroxide form, and then this basic solution is neutralized by the addition of an adequate acid solution. The main issues associated with using this method with antibiotics are their poor solubility in most common solvents and the instability of the antibiotics in the presence of strong bases. To overcome these issues, Ferraz et. al. (2012) developed the buffer neutralization method [66]. In fact, ILs containing ampicillin were prepared with good yields by using this method [66]. In this work, in regard to their particular physical-chemical properties, such as their low melting point, very high water solubility, as well as their biocompatibility and low toxicity, it was determined that the choline cation paired with ampicillin presented the best results [66,71].

Another example using beta-lactam antibiotics is the use of penicillin and amoxicillin by the same authors [36]. This work highlighted that organic salts and ionic liquids that contain ammonia hydrolysates of amoxicillin and penicillin could be used as new antibacterial agents. Also, the authors emphasized that the focus of the new antibacterial agent ILs should not be only on the toxicity and hydrophobicity of the counter ion but also on the outcome [36].

Florindo et. al. (2014) [72] produced ILs based on fluoroquinolones, a different group of antibiotics. Their main objectives were as follows: (a) to create a viable synthetic method; (b) to create ILs with relevant pharmacological properties; (c) to achieve a balance between the water solubility and the lipophilicity of the ILs; (d) to demonstrate that a variety of behaviors can be tuned with the right cation. The new ILs improved their solubility in water and in simulated biological fluids at 25 °C [72].

ILs as antimicrobial agents are not only based on antibiotics; Ferraz et al. (2016) produced other examples of ILs as antimicrobial agents—ILs based on antimalarial parasite drugs [73]. In this work, they described a new low-cost and efficient method to produce six ILs based on primaquine, an antimalarial drug, and evaluated in vitro the performance of these ILs against three stages of malaria parasites [73]. These new ILs were found to display similar or better in vitro activities than their covalent analogues, *N*-cinnamoyl-primaquine derivatives, which had been formerly developed [74,75,76], and to the parent drug. This line of work opens a new pathway to novel low-cost antimalarial IL leads, which are of undeniable importance for the chemoprophylaxis, radical cure, and containment of malaria [33,73]. Since these ILs showed better results than those of the respective covalent (amide) analogues and of the parent primaquine [73], the authors hypothesized that such behavior might be due to an enhanced ability of the ionic compounds to permeate into *Plasmodium*-infected erythrocytes; as such, a differential scanning calorimetry-based study of the interactions between the ionic liquids and membrane models was tested [74]. The work showed that, at the molecular level, the primaquine-derived ILs may contribute to an increased permeation of the parent drug into malaria-infected erythrocytes, which has relevant implications towards novel antimalarial approaches based on ILs [74].

When we use ILs with long alkyl chains, there is a tendency for these compounds to aggregate in solution, forming amphiphilic micelles and displaying surface activity [33,77,78,79,80]. Until now, some works have shown that ILs can display broad activity spectra, affecting both Gram-positive and Gram-negative bacteria as well as mycobacteria and fungi [77,78,79,80,81].

Additionally, 1-alkyl-3-methylimidazolium fumarates also showed antimicrobial potential against Gram-positive *Bacillus subtilis*, Gram-negative *Escherichia coli*, and the yeast *Saccharomyces cerevisiae* [82]. Moreover, the antimicrobial properties of phosphonium and ammonium ILs have also been studied [31]. Ammonium cations combined with azolate anions, as well as hydroxylammonium-based ILs, were found to have antibacterial and antifungal properties, with the latter actually presenting activity against relevant human pathogens such as *Staphylococcus aureus*, *Salmonella typhi*, and *Vibrio cholera* [31,83,84]. Other studies on bioactive phosphonium-based ILs have also reported on the toxicity of those ILs within the context of the food industry [85]. Finally, it was found that triphenylamine phosphonium ILs can spontaneously adopt nanostructures displaying activity against Gram-positive bacteria like *S. aureus*. [31,86], whereas other studies showed that diphosphonium-based ILs exhibit a broad spectrum of antimicrobial activity against ocular pathogens [31,87].

In Table 1, the main ILs described as antimicrobial agents are presented.

#### ILs Active Against Resistant Microorganisms

Antimicrobial resistance is a serious issue, so the development of ILs based on antibacterial agents must have this in mind. In Table 1, the papers with an asterisk have tested the ILs on resistant strains. For example, ILs based on ampicillin showed growth inhibition and bactericidal properties on some Gram-negative resistant bacteria when compared to the sodium ampicillin and the initial bromide and chloride salts [91]. The authors used clinically isolated resistant strains, such as *E. coli* TEM CTX M9, *E. coli* CTX M2, and *E. coli* AmpC MOX. The results showed a high relative decrease of the inhibitory concentration (RDIC) values of [C16Pyr][Amp], especially against two resistant Gram-negative strains *E. coli* TEM CTX M9 (RDIC > 1000) and *E. coli* CTX M2 (RDIC > 100), thus clearly demonstrating the potentially promising role of API–ILs as antimicrobial drugs, particularly against resistant bacterial strains [91].

Another example from this group is ILs and organic salts (OSILs) that contain anionic penicillin G and amoxicillin hydrolysate derivatives [36] In this case, the new compounds revealed a relative decrease of inhibitory concentrations. These in vitro results indicate that a basic transformation of the classic antibiotics into the hydrolyzed organic salts can considerably change the activity of a drug, including giving rise to potent formulations of antibiotics against deadly resistant strains of bacteria [36].

Cole et al. [63] also proposed recycling antibiotics with ILs or organic salts. In their work, they used ampicillinate, carbenicillinate, cephalothinate, and oxacillinate as anions and tested against resistant strains, with their results showing a promising approach to combatting antibiotics resistance [63].

Thus, several works describe the success of using biocompatible organic cations, such as choline, alkylpyridiniums, and alkylimidazoliums (Table 1), in combination with inorganic anions or antibiotics.

### 2.5. Poly(ionic Liquids)

Poly(ionic liquids), or polymerized ionic liquids (PILs), are a recent class of ILs [99,100]. PILs can self-assemble into polymeric nanoparticles with highly ordered inner structures [101]. Other characteristics, like their versatility and capacity to be adjusted into different morphologies, sizes, and surface charges makes them interesting for antibacterial agents or systems [100]. Despite the rapid growth in the applications of PILs, more studies are needed to compare the advantages and disadvantages of PILs against ILs, such as the work of Zhen et al. [102]. The authors studied imidazolium-type IL monomers and their corresponding PILs and poly(ionic liquid) membranes on the antimicrobial activities against both *E. coli* and *S. aureus* in the attempt to find a relation between the small molecules and homopolymers and the corresponding polymer membranes [102].

## 3. Conclusions

Considering the unique properties of ILs, it is not surprising that in recent years there has been significant interest in their potential applications in antibacterial research, not only for new antibiotic synthesis but also as a potential means to reformulate existing antibiotics. In recent years, there has been a growing global interest in ILs, with studies describing their characteristics, sometimes detailing their unique chemical and physical properties, exploring “task-specific ILs” or those based on APIs, and researching their biological activities [33,50].

To counteract the disadvantages of solid drugs, new ILs could be linked with pharmacologically active ingredients, forming API-ILs, an alternative to common crystalline salts [24,27,28,31,38,40,43]. The possibility to eliminate or reduce the negative side effects of an active compound by delivering it as an API–IL is attractive for pharmaceutical, environmental, and medical applications. The combination of a specific IL counterion with a specific API can influence the toxicity, biodegradability behavior, water-solubility, permeability, and drug formulation process, and can change some of the biological properties of the API [36,38,71,74,91,103,104]. In the next few years, it is expected that the number of novel API-ILs will greatly increase owing to the interest in the topic as well as the significant advantages of having pharmaceutical drugs as organic salts or ILs [28,31,38]. The use of API-ILs appears to be a very promising pharmaceutical strategy. Nevertheless, some drawbacks of the API-ILs concept should also be mentioned. The API-IL strategy cannot be considered the ultimate solution to the polymorphism problem, because polymorphs of API-ILs have already been described in literature [105]. An ideal API-IL should be liquid with a limited formation of polymorphs and should keep the level of activity exhibited by the parent drug [38,106,107].

**Figure 3:**
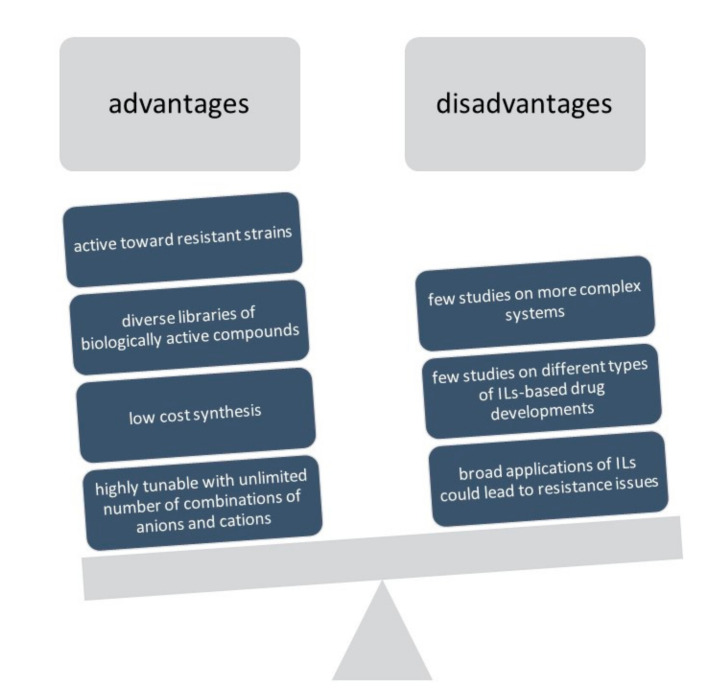
Advantages and disadvantages of the use of ILs as antimicrobial agents, adapted from Ferraz et. al. (2018) [28].

This study highlights that ILs as bioactive agents may have some advantages and some disadvantages [28], as presented in Figure 3. The main advantages are that this kind of compound can be easily tunable and be produced with an unlimited number of combinations of anions and cations. They can be synthesized with a low budget and they may allow for the creation of diverse libraries of biologically active compounds. Finally, they could be a way to fight antimicrobial resistance. One of the disadvantages described here is that there have been few studies about them. Their mechanisms of action are still unknown, and broad applications of ILs could lead to resistance issues. Also, there are still few studies on different types of ILs-based drug developments and there are also few studies on more complex systems.

Nevertheless, ILs combined with antibiotics could be a “weapon” by themselves or combined with phage therapy and lysine therapy. This future approach may be able to fight one of the biggest problems currently facing the world: uncontrolled infections, including those resulting from antimicrobial resistance.

## Figures and Tables

**Table 1:** Brief summary of ILs with antimicrobial activity.

ILs with Antimicrobial Activity		Microorganisms	Authors and Year of Publication
Cation	Anion		
didecyldimethylammonium, benzalkonium	lactate	*Micrococcus luteus,* *Staphylococcus aureus,* *Staphylococcus epidermidis,* Streptococcus *mutans,* *Enterococcus faecium,* *Moraxella catarrhalis,* *Escherichia coli,* *Serratia marcescens,* *Proteus vulgaris,* *Pseudomonas aeruginosa,* *Bacillus subtilis*	Cybulski, 2008 [58]
didecyldimethylammonium, benzalkonium, cetylpyridinium, 3-hydroxy-1-octyloxymethylpyridinium	saccharinate, acesulfamate	*Staphylococcus aureus,* *Enterococcus faecium,* *Escherichia coli,* *Micrococcus luteus,* *Staphylococcus epidermidis,* *Klebsiella pneumoniae*	Hough-Troutman 2009 * [88]
didecyldimethylammonium, benzalkonium, domiphen	mandelate, prolinates	*Micrococcus luteus,* *Staphylococcus aureus,* *Enterococcus faecium,* *Serratia marcescens,* *Proteus vulgaris,* *Pseudomonasaeruginosa,* *Bacillus subtilis*	Cybulski, 2011 [89]
1-alkyl-3-methylimidazolium, alkylpyridinium	chloride, bromide	*Escherichia coli,* *Staphylococcus aureus,* *Bacillus subtilis*	Yu, 2011 [90]
cetylpyridinium, 1-hexadecyl-3-methylimidazolium, 1-hexadecyl-2,3-dimethylimidazolium	ampicillinate	*Escherichia coli,* *Klebsiella pneumoniae,* *Staphylococcus aureus,* *Enterococcus faecium*	Cole, 2011 [69]
1-ethyl-3-methylimidazolium, 1-hydroxy-ethyl-3-methylimidazolium, choline, tetraethylammonium, cetylpyridinium, trihexyltetradecylphosphonium.	ampicillinate	*Escherichia coli,* *Klebsiella pneumoniae,* *Staphylococcus aureus,* *Enterococcus faecali,* *Staphylococcus epidermidis*	Ferraz, 2014 * [91]
chlorhexidine	ampicillinate, carbenicillinate, cephalothinate, oxacillinate	*Escherichia coli,* *Klebsiella pneumoniae,* *Pseudomonas aeruginosa,* *Staphylococcus aureus,* *Streptococcus faecalis,* *Bacillus cereus* *Enterococcus faecium*	Cole, 2015 * [68]
1-alkyl-3-methylimidazolium, trimethylalkylammonium, tributylmethylammonium, trioctylmethylammonium, tributylmethylphosphonium, trioctylmethylphosphonium, 1-ethyl-3-methylpiperidinium, 1-ethyl-1-methylmorpholinium, 1-butyl-3-methylpyrrolidinium	nalidixate	*Salmonella* species	Mester, 2016 * [92]
3-cinnamyl-1-alkyl-imidazolium	chloride	*Staphylococcus aureus,* *Streptococcus pyogenes,* *Staphylococcus epidermidis,* *Escherichia coli,* *Pseudomonas aeruginosa,* *Acinetobacter baumannii*	Doria, 2018 [59]
*N*-arylalkyl pyrimidinium, *N*-aryloxyalkylpyrimidinium	bromide, tetrafluoroborate, bis(trifluoromethanesulfonyl)amide	*Staphylococcus aureus,* *Bacillus pumilis,* *Bacillus* *subtilis,* *Escherichia coli,* *Klebsiella pneumonia,* *Pseudomonas aeruginosa*	Goel, 2019 [93]
pyridoxinium O,O-2-isopropyl-5-methylcyclohex-1-yl], pyridoxinium O,O-2-isopropyl-5-methylcyclohex-1-yl], pyridoxinium O,O-di(2-isopropyl-5-methylphenyl. pyridoxinium O,O-1,3,3-trimethylbicyclo [2.2.1]-hept-2-yl], nicotinium O,O- 2-isopropyl-5-methylcyclohex-1-yl], nicotinium O,O-di(2-isopropyl-5-methylphenyl), nicotinium O,O-di[endo)-1,7,7-trimethylbicyclo[2.2.1]hept-2-yl, nicotinium O,O-di[2,6,6-trimethylbicyclo[3.1.1]-hept-3-yl], nicotinium O,O-dibutyl, Nicotinamide	dithiophosphate,	*Pseudomonas aeroginosa* *Klebsiella pneumoniae,* *Staphylococcus aureus,* *Staphylococcus epidermis*	Dang, 2019 * [94]
3-(3-propylimidazole)-1,8-naphthalene monoimide-1-dodecylimidazolium, 3-(3-propylimidazole)-1,8-naphthalene monoimide-1-hexacylimidazolium	bromide, bis(trifluoromethane)sulfonamide	*Staphylococcus aureus,* *Escherichia coli,* *Pseudomonasaeruginosa* *Enterococcus faecali*	Duman, 2019 [95]
*N*-cinnamoylimidazolium	bromide	*Staphylococcus aureus,* *Staphylococcus epidermidis,* *Acinetobacter baumannii,* *Pseudomonasaeruginosa*	Forero-Doria, 2019 [60]
3-methyl-1-alkylimidazolium, 3-methy-1-alkyllimidazolium-furanchalcone hybrid	bromide, tetrafluoroborate, hydroxide	*Escherichia coli,* *Pseudomonas aeruginosa,* *Staphylococcus aureus,* *Bacillus cereus,* *Streptococcus mutans,* *Streptococcus agalactiae,* *Bacillus subtilis*	Arque, 2020 [96]
1-hexadecyl-3-methylimidazolium, 1,10-bis(methylimidazolium-1-yl) decane, 1-hexadecyl-2,3,4,5-tetram	chloride, bromide, n-hexadecyl, methanesulfonate	*Dickeya chrysanthemi;* *Escherichia coli;* *Erwinia psidii;* *Pectobacterium carotovorum,* *Pseudomonas syringae,* *Xanthomonas axonopodis*	Neves, 2020 [97]
1-butyl-3-methylimidazolium, [2-(4-hydroxyethoxy)ethyl]-3-methylimidazolium, 1-(3-hydroxypropyl)-3-me-thylimidazolium, imidazolium	salicylate	*Staphylococcus aureus,* *Bacillus subtilis.* *Enterococcus faecalis,* *Proteus mirabilis,* *Escherichia coli,* *Pseudomonas aeruginosa*	Jovanović-Šanta, 2020 * [98]
Tetraethylammonium, trihexyl(tetradecyl)phosphonium, cetylpyridinium, 1--ethyl--3--methylimidazolium, 3--(2--hydroxyethyl)--1--methylimidazolium, choline,	penicillin hydrolysate, amoxicillin hydrolysate	*Escherichia coli,* *Staphylococcus aureus*	Ferraz, 2020 * [36]

* In these works, resistant strains were also studied.
